# Supplementary material for: Differential Reinforcement without Extinction: An Assessment of Sensitivity to and Effects of Reinforcer Parameter Manipulations
Source: Behav Sci (Basel). 2024 Jun 28;14(7):546. doi: 10.3390/bs14070546 (PMC11274137; doi:10.3390/bs14070546)
Supplement: Supplementary file 1 [file behavsci-14-00546-s001.zip › behavsci-3033237-supplementary.pdf]

Social Validity Questionnaire: Parent

1= strongly disagree, 2= somewhat agree, 3= mostly agree, 4= strongly agree

1. Based on the two videos you just saw:
    a. Did you observe a decrease in challenging behavior from video A to video B?

       1 2 3 4

    b. Did you observe an increase in communication from video A to video B?

       1 2 3 4

2. Do you feel that the intervention was appropriate given your child's age and behavior?

   1 2 3 4

3. Would you recommend this intervention to others?

   1 2 3 4

4. Do you have any additional comments?

____

____

____
